# Sweet pepper extract reduces fat storage in *Caenorhabditis elegans* by SREBP‐SCD axis based on multiomics analysis

**DOI:** 10.1002/fsn3.4266

**Published:** 2024-06-14

**Authors:** Junyi Wang, Peng Xu, Xinhua Liu, Chunxin Cao, Yingkun Sheng, Jianfeng Wang

**Affiliations:** ^1^ Life Sciences Zhejiang Normal University Jinhua China; ^2^ Hangzhou Normal University of Basic Medicine Hangzhou China; ^3^ Jinhua Academy of Agricultural Sciences Jinhua China; ^4^ Xingzhi College Zhejiang Normal University Jinhua China

**Keywords:** *Caenorhabditis elegans*, fat accumulation, multiomics analysis, SREBP‐SCD axis, sweet peppers

## Abstract

Sweet pepper, a globally commercialized horticultural crop, has been demonstrated to impede fat accumulation, but its mechanism remains incompletely understood. This study was designed to explore the potential mechanism of sweet pepper in reducing fat accumulation in *Caenorhabditis elegans* through RNA‐seq and metabolome analysis. A total of 22 metabolites were identified from sweet pepper by UHPLC‐ESI‐TOF‐MS analysis. In vivo, sweet pepper significantly inhibited α‐glucosidase activity and reduced the levels of glucose, triglycerides (TG), total cholesterol (TC), and the area stained with oil red O. Additionally, it increased body length and the number of head swings in *C. elegans* compared to the control group. The KEGG enrichment analysis revealed significant enrichment of the biosynthesis of unsaturated fatty acids signaling pathway among the differentially expressed genes and metabolites. Furthermore, the mRNA levels of sterol regulatory element‐binding proteins (SREBPs) ortholog SBP‐1, as well as the stearyl CoA desaturase‐1 (SCD1), including *fat‐5*, *fat‐6*, and *fat‐7*, were significantly decreased after treatment with sweet pepper. Collectively, sweet pepper effectively reduces fat accumulation, which is probably related to downregulating the SREBP‐SCD axis, offering new insights for future functional food development.

## Graphical abstract

A new sweet pepper variety, initially named Jinjiao 23–6, effectively reduces fat accumulation in *C. elegans*. Sweet pepper alleviates fat accumulation in *C. elegans* by modulating the synthesis of unsaturated fatty acids through SREBP‐SCD axis.

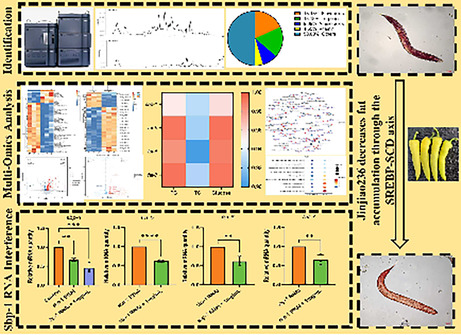

## 1 INTRODUCTION

Type 2 diabetes mellitus (T2DM) is a metabolic disorder characterized by hyperglycemia and insulin resistance. The incidence of T2DM is increasing globally (Abuyassin & Laher, 2016). Obesity is the predominant risk factor for nonalcoholic fatty liver disease (NAFLD), T2DM, and cardiovascular disease (CVD), contributing to a global rise in cardiovascular morbidity and mortality (Dahik et al., 2020; La Sala & Pontiroli, 2020). Excessive fat accumulation in hepatocytes initially causes obesity, which then progresses to NAFLD, damaging liver function and potentially leading to irreversible liver fibrosis and liver cancer (Bessone et al., 2019; Kang et al., 2022). Jodok Fink and colleagues reported that individuals with severe obesity (BMI ≥40 kg/m^2^) have a 7.4 times higher risk of developing T2DM compared to those with normal weight (Fink et al., 2022). Fat accumulation and adipose tissue inflammation are typical characteristics of obesity, which is widely recognized as an important pathological factor in the development of T2DM, insulin resistance, and CVD (He et al., 2021; Kolb, 2022; Ruze et al., 2023). Therefore, reducing fat accumulation is crucial in preventing obesity, T2DM, NAFLD, and CVD. Stearyl CoA desaturase‐1 (SCD1) is an essential enzyme that regulates the transformation of saturated fatty acids (SFA) into monounsaturated fatty acids (MUFA), and plays a crucial role in controlling fatty acid metabolism and the balance between SFA and MUFA (Balatskyi & Dobrzyn, 2023). Activated SCD1 promotes MUFA biosynthesis, fat accumulation, and the development of T2DM (Igal & Sinner, 2021; Tibori et al., 2022, 2024). Recently, multiple studies have demonstrated that reducing the expression levels of *sbp‐1*, *fat‐5*, *fat‐6*, and *fat‐7* significantly decreases intestinal fat accumulation in *C. elegans* (Bai et al., 2021; Nomura et al., 2010). These data imply that suppression of the SBP‐1‐SCD1 axis may reduce fat accumulation.

Sweet peppers are rich in functional substances such as capsaicin, flavonoids, polyphenols, and vitamins (Sanatombi, 2023). Research indicates that capsaicin, as the primary active ingredient, can accelerate metabolism, increase energy expenditure, promote fat burning, and lower blood sugar levels (Varady et al., 2022; Whiting et al., 2014; Zsiborás et al., 2018). Furthermore, investigations have found a significant increase in plasma glucagon‐like peptide levels after incorporating a certain amount of capsaicin into meals (Smeets & Westerterp‐Plantenga, 2009). Animal studies also indicate that capsaicin can activate TRPV1, reducing body weight and blood lipids, and improving visceral fat (Chen et al., 2015). Red pepper leaf extract suppressed the expression of SREBP‐1, PPAR‐γ in the liver, leading to reduced fat accumulation in mice fed a high‐fat diet (Oh et al., 2023). However, whether sweet pepper reduces fat accumulation in *C. elegans* through the SREBP‐SCD axis has not been previously reported.

Therefore, this study employed metabolomics and RNA‐seq analysis to investigate the correlation between the reduction in fat accumulation, and glucose content and the SREBP‐SCD axis in *C. elegans* after treatment with sweet pepper, providing new insights for the future development of functional foods.

## 2 MATERIALS AND METHODS

### 2.1 Reagents and materials

*Caenorhabditis elegans* and *E. coli* OP50 were acquired from the Caenorhabditis Genetics Center (CGC, USA). The following reagents were utilized in the experiments: agar, KH_2_PO_4_, Na_2_HPO_4_, NaH_2_PO_4_, NaCl, NaClO, CaCl_2_, NaOH, MgSO_4_, cholesterol, yeast extract, tryptone, peptone, acetonitrile (liquid phase), formic acid (liquid phase), animal total RNA isolation kit, and glucose content assay kit were sourced from Sangon Biotech (Shanghai, China). Trichloromethane, ethyl alcohol, 1‐butanol, and hydrochloric acid were purchased from Lanxi ShenYing (Jinhua, China). TB Green® Premix Ex Taq™ and PrimeScript™ RT reagent kit with gDNA Eraser were supplied by Takara (Beijing, China). Commercial kits for measuring glucose, triglycerides (TG), and total cholesterol (TC) were provided by the Nanjing Institute of Biological Engineering (Nanjing, China). A modified oil red O staining kit was obtained from Beyotime Biotechnology Co., Ltd. (Shanghai, China).

The nematode growth culture medium was formulated with the following components: 2.5 g peptone, 3.0 g NaCl, 0.111 g CaCl_2_, 0.12 g MgSO_4_, 0.005 g cholesterol, 3.4 g KH_2_PO_4_, and 17.0 g agar.

### 2.2 Screening of sweet pepper varieties

Through selective breeding and α‐glucosidase activity screening (Figure S1), a new sweet pepper variety with desirable overall characteristics was obtained, initially named Jinjiao 23‐6. Plant specimens are preserved at the Southern Agriculture and Forestry Resources Laboratory of Xingzhi College, Zhejiang Normal University.

### 2.3 Sample preparation and UHPLC‐MS analysis for metabolomics

The extract was prepared from lyophilized sweet pepper by dissolving the powder in deionized water at a ratio of 1:5 (m/V). The solution underwent oscillatory extraction at 4°C for 2 h. Subsequently, the supernatants were collected, filtered, and lyophilized.

The extract was dissolved in ultrapure water by sonication and centrifuged for 10 min at 12,000 × g. UHPLC was conducted on the supernatant using AB SCIEX™ (Nexera UHPLC LC 30A, SHIMADZU, Suzhou, China; TripleTOF5600+, AB SCIEX™, Redwood City, CA, USA).

The ChromCore 120 C18 column was used in this study and the LC‐MS conditions were as follows: column temperatre was 40°C, monile phase was 0.1% formic acid (A), 100% acetonitrile (B), flow rate was 0.3 mL/min.

An isocratic elution of 5% B to 70% B lasted from 0 to 10 min; 70% B to 100% B at 10–17 min, and 100% to 5% B at 17–21 min.

Electrospray ionization (ESI) positive‐ and negative‐ion modes of electrospray ionization were used. The ESI source conditions are as follows: ion source gas1 (Gas 1): 50, ion source gas2 (Gas 2): 50, curtain gas (CUR): 25, source temperature: 500°C (positive ion) and 450°C (negative ion), ion spray voltage floating (ISVF) 5500 V (positive ion) and 4400 V (negative ion), TOF MS scan range: 100–1200 Da, product ion scan range: 50–1000 Da, TOF MS scan accumulation time: 0.2 s, and product ion scan accumulation time: 0.01 s. Secondary mass spectra were acquired using a high‐sensitivity mode with information‐dependent acquisition; de‐clustering potential was ±60 V and the collision energy was 35 ± 15 eV.

The acquired data were preprocessed using the software MS‐DIAL 4.70 (Tsugawa et al., 2015); peak extraction, noise removal, deconvolution, and peak alignment were conducted to generate a 3D data matrix in CSV format. The extracted peak information was compared using three libraries: MassBank, Respect, and GNPS.

### 2.4 Culture and treatment of *C. elegans*

The *C. elegans* were cultured at 20°C in nematode growth media (NGM) with OP50 serving as the food source. To synchronize the populations of *C. elegans*, the worms were treated with sodium hypochlorite. The protocols for cultivation and maintenance followed those outlined in the WormBook (Stiernagle, 2006). Subsequently, *C. elegans* nematodes were cultured at 20°C for 48 h in media containing water, 0.5 mg/mL, 0.75 mg/mL, and 1 mg/mL of sweet pepper.

### 2.5 Body length and head swing analysis

The synchronized *C. elegans* (L1) were added to NGM media containing different concentrations of sweet pepper extract and cultured at 20°C for 48 h.

The body length and head swing of *C. elegans* were measured using a Mshot ML31 biomicroscope with a MShot Image Analysis System (Guangzhou Micro‐shot Technology Co., Guangzhou, China). The Student's *t*‐test was used for statistical analysis. The presented data are expressed as mean ± SD.

### 2.6 Biochemical assays

Synchronized L4‐stage *C. elegans* were transferred onto NGM culture medium containing 1 mg/mL of sweet pepper extract. A total of 1500 *C. elegans* were selected and washed with M9 to remove residual *E. coli*. After diluting to the same concentration with deionized water and grinding thoroughly, glucose, TC, and TG levels were measured in strict accordance with commercial kit instructions. The Student's *t*‐test was used for statistical analysis. The presented data are expressed as mean ± SD.

### 2.7 Oil red O staining

After exposure to 1 mg/mL sweet pepper extract, 1‐day‐old *C. elegans* were treated with 4% paraformaldehyde, subjected to three cycles of freeze‐thawing, centrifuged, and washed with M9 solution to eliminate any remaining fixative. Subsequently, *C. elegans* were stained by using modified oil red O staining kit (Beyotime, Shanghai, China). The worms were placed on a 1% agar pad and observed using a Mshot ML31 biomicroscope with an MShot Image Analysis System (Guangzhou Micro‐shot Technology Co, Guangzhou, China). The oil red O staining area was quantified by using Image J software. The Student's *t*‐test was used for statistical analysis. The presented data are expressed as mean ± SD.

### 2.8 Transcriptome sequencing analysis

The gene expression profiles of *C. elegans* treated with 1 mg/mL of sweet pepper extract for 1 day and untreated *C. elegans* were obtained through RNA seq. Firstly, total RNA was extracted using TRIzol reagent following the manufacturer's protocol. The RNA purity and quantity were assessed using a NanoDrop 2000 spectrophotometer (Thermo Scientific, USA), and the RNA integrity was evaluated with an Agilent 2100 Bioanalyzer (Agilent Technologies, Santa Clara, CA, USA). Subsequently, a transcriptome library was constructed using the VAHTS Universal V5 RNA‐seq Library Prep Kit as per the instructions.

Sequencing of the library was performed on the Illumina Novaseq 6000 platform. The raw reads in fastq format were processed using fastp software (Chen et al., 2018) for subsequent data analysis. Alignment to the *C. elegans* reference genome was conducted using HISAT2 software (Kim et al., 2015) to calculate gene expression levels (FPKM) (Roberts et al., 2011). DESeq2 software was utilized to conduct differential expression gene (DEG) analysis, with DEGs defined as genes having a *q*‐value <0.05 and a fold change >2 or <0.5 (Love et al., 2014). Finally, the function of DEGs was annotated by Gene Ontology (GO) and Kyoto Encyclopedia of Genes and Genomes (KEGG) enrichment analysis. The RNA libraries were sequenced by OE Biotech, Inc., Shanghai, China.

### 2.9 Untargeted metabolomics analysis

The samples of *C. elegans* treated with sweet pepper extract and untreated *C. elegans* were collected to further explore the metabolic changes. An UHPLC‐Q‐Exactive‐Orbitrap triple quadrupole mass spectrometer (Waters, USA) equipped with a Waters ACQUITY UPLC HSS T3 liquid chromatography column (2.1 × 100 mm, 1.7 μm) was used for this study. The column temperature was 45°C and the injection volume was 3 μL. A binary solvent system was used, in which eluent A consisted of 0.1% formic acid water and eluent B of acetonitrile, using the following gradient elution: 0–2 min (5% B), 2–4 min (5%–30% B), 4–8 min (30%–50% B), 8–10 min (50%–80% B), 10–14 min (80%–100% B), 14–15 min (100%–100% B), 15–15.1 min (100%–5% B), and 15.1–16 min (5% B).

The mass spectra were acquired ranging from −70 to 1050 mass‐to‐charge ratio with a primary resolution of 70,000 and a secondary resolution of 17,500. The sheath gas flow rate was 35, and the auxiliary gas flow rate was 8. The spray voltages were set at 3.8 KV (positive ion) and 3 KV (negative ion). The capillary temperature was maintained at 320°C, and the auxiliary gas heater temperature was at 350°C. Progenesis QI v3.0 software (Nonlinear Dynamics, Newcastle, UK) was used for baseline filtering, peak recognition, integration, retention time correction, peak alignment, and normalization of the original data.

Metabolites were identified based on multiple dimensions including retention time (RT), accurate mass, secondary fragmentation, and isotope distribution using The Human Metabolome Database (HMDB), Lipidmaps (v2.3), METLIN database, and the LuMet‐Animal3.0 local database. Statistical significance (*p*‐value) was computed using a single‐factor analysis (*t*‐test). Metabolites with VIP > 1 and a *p*‐value <.05 were considered differentially expressed metabolites (DEMs). Pathway enrichment analysis of differential metabolites was performed using the KEGG database and applied hypergeometric testing to identify significantly enriched pathway entries compared to the entire background within the significantly differentially expressed metabolites. The metabolites were analyzed by OE Biotech, Inc., Shanghai, China.

### 2.10 Real‐time quantitative PCR

The RNAiso Plus (Takara, Dalian, China) was used to extract total RNA from *C. elegans*. Worms were ground with 1 mL RNAiso Plus, in an ice bath, and the RNA was extracted with 200 μL chloroform for 5 min. After the addition of one‐third ethanol and centrifugation at 12,000 × g for 15 min, the precipitate was washed with 70% ethanol. RNA integrity was checked using 1% agarose gel electrophoresis. The possible gDNA present in the extracted RNA was removed using the PrimeScript™ RT reagent kit with gDNA Eraser kit (Takara, Dalian, China) and the RNA was reverse transcribed into cDNA using the PrimeScript™ RT reagent kit with gDNA Eraser and T100 Thermal Cycler (Bio‐Rad, USA).

Real‐time quantitative PCR was performed on cDNA from *C. elegans* treated with 1 mg/mL sweet pepper extract, using TB Green® Premix Ex Taq™ (Takara, Dalian, China). The relative gene expression was calculated by the 2^−ΔΔCt^ method. Actin‐1 was chosen as the housekeeping gene. Primers are shown in the Table S1.

### 2.11 RNA interference

The *sbp‐1* RNAi *E. coli* strain was revived from the interference library and cultured on NGM medium supplemented with 100 mM IPTG and 50 μg/mL ampicillin at 37°C for 12 h. Afterward, synchronized *C. elegans* at the L4 stage were transferred to this medium containing 1 mg/mL sweet pepper extract and cultured at 20°C for 48 h. Additionally, the levels of TC, TG, and the relative gene expression of *sbp‐1*, *fat‐5*, *fat‐6*, and *fat‐7* were measured.

## 3 RESULTS

### 3.1 Component analysis

**FIGURE 1:**
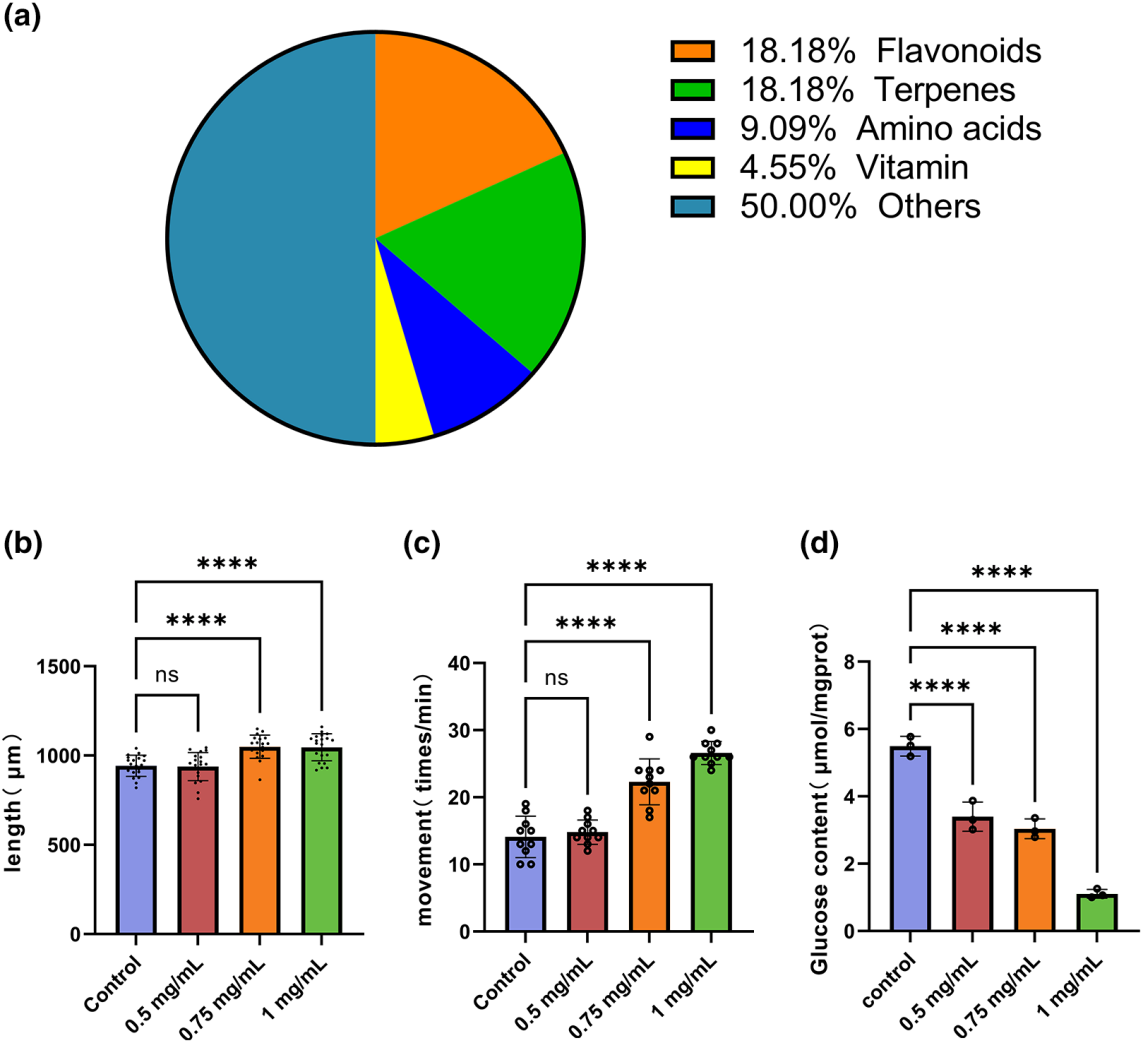
Chemical composition of sweet pepper and its effect on the behavior of *C. elegans*. (a) In the extract, we identified seven types of flavonoids, five types of terpenes, three types of amino acids, and one type of alkaloid. (b) Body length of *C. elegans* at different concentrations (*n* = 20). (c) Head swing of *C. elegans* at different concentrations (*n* = 10). (d) Glucose content in *C. elegans* at different concentrations (*n* = 3). Statistical analysis was conducted using Student's *t*‐test. Data are shown as mean ± SD. ****p < .0001, ns, no significance.

A total of 88 ingredients were identified in sweet pepper using UHPLC‐ESI‐TOF‐MS (Figure S2), primarily consisting of terpenoids, alkaloids, flavonoids, and amino acids (Figure 1a). After a detailed comparison of the mass‐to‐charge ratios (m/z) and secondary fragment information, 22 compounds were specifically identified and are listed in Table 1.

**TABLE 1:** Pepper extract composition.

Identified	RT (min)	Precursor m/z	Reference m/z	Error (ppm)	Adduct ion	Formula	Ontology
Methyl gallate	1.265	185.0396	185.03999	−2.120	[M+H]+	C_8_H_8_O_5_	Galloyl esters
Pyridoxine	1.624	170.0592	170.0590	1.420	[M+H]+	C_8_H_11_NO_3_	Vitamin
5‐Hydroxy‐6,7‐dimethoxyflavone	3.123	297.1584	297.1581	0.973	[M−H]−	C_17_H_14_O_5_	Flavonoids
[(2S,3R,4S,5S,6R)‐3,4,5‐Trihydroxy‐6‐(hydroxymethyl)oxan‐2‐yl] (3aS,6Z,10E,11aR)‐10‐methyl‐3‐methylidene‐2‐oxo‐3a,4,5,8,9,11a‐hexahydrocyclodeca[b]furan‐6‐carboxylate	4.832	463.1364	463.1365	−0.165	[M+H]+	C_21_H_28_O_9_	Terpenes
4‐amino‐1‐((2R,3R,4S,5R)‐3,4‐dihydroxy‐5‐(hydroxymethyl)tetrahydrofuran‐2‐yl)pyrimidin‐2(1H)‐one	4.832	266.0703	266.0700	1.193	[M+H]+	C_9_H_13_N_3_O_5_	Pyrimidine nucleosides
Caperatic acid	5.082	401.2227	401.2226	0.249	[M−H]−	C_21_H_38_O_7_	Tricarboxylic acids and derivatives
(2R,3R,4S,5S,6R)‐2‐[6‐Hydroxy‐3‐[(E)‐3‐hydroxybut‐1‐enyl]‐2,4,4‐trimethylcyclohexyl]oxy‐6‐(hydroxymethyl)oxane‐3,4,5‐triol	5.267	429.1886	429.1885	0.231	[M+H]+	C_19_H_34_O_8_	Terpenes
Methyl 2‐[[3‐[(3,3‐dimethyloxiran‐2‐yl)methyl]‐4‐hydroxyphenyl]methyl]‐4‐hydroxy‐3‐(4‐hydroxyphenyl)‐5‐oxofuran‐2‐carboxylate	5.664	463.1363	463.1363	−0.045	[M+H]+	C_24_H_24_O_8_	Fatty acid esters
Dolasetron	6.086	323.1400	323.1401	−0.340	[M−H]−	C_19_H_20_N_2_O_3_	Indole carboxylic acids and derivatives
[(2R,3S,4S,5R,6S)‐3,4‐dihydroxy‐6‐[3,7,8‐trihydroxy‐2‐(4‐hydroxy‐3‐methoxyphenyl)‐4‐oxochromen‐5‐yl]oxy‐5‐[(2S,3R,4R,5R,6S)‐3,4,5‐trihydroxy‐6‐methyloxan‐2‐yl]oxyoxan‐2‐yl]methyl acetate	6.126	681.3514	681.3510	0.660	[M−H]−	C_30_H_34_O_18_	Flavonoids
4,4′‐Dimethoxydalbergione	6.440	283.0977	283.0976	0.352	[M−H]−	C_17_H_16_O_4_	Dalbergiones
8‐(3‐Methoxy‐2‐(methoxycarbonyl)phenyl)octanoic acid	6.600	307.1544	307.1540	1.331	[M−H]−	C_17_H_24_O_5_	O‐methoxybenzoic acids and derivatives
Pomiferin	7.431	419.2502	419.2502	0.048	[M−H]−	C_25_H_24_O_6_	Flavonoids
9‐Methoxy‐4,4‐dimethyl‐13‐phenyl‐6,14‐dioxatetracyclo[8.4.0.02,7.03,5]tetradeca‐1,7,9‐trien‐11‐one	7.665	335.1351	335.1351	0.089	[M−H]−	C_21_H_20_O_4_	Flavonoids
MGMG 18:3	7.665	513.3046	513.3047	−0.175	[M−H]−	C_27_H_46_O_9_	Lipids
(1S,2R,7S,13R,14R,16S,19S,20S)‐19‐(Furan‐3‐yl)‐11‐hydroxy‐9,9,13,20‐tetramethyl‐4,8,15,18‐tetraoxahexacyclo[11.9.0.02,7.02,10.014,16.014,20] docos‐10‐ene‐5,12,17‐trione	8.372	483.1661	483.1661	0.103	[M−2H]2−	C_26_H_28_O_9_	Steroid lactones
Galdosol	8.529	343.2281	343.2279	0.465	[M−H]−	C_20_H_24_O_5_	Terpenes
Piscidic acid	9.350	255.1332	255.1334	−0.586	[M−H]−	C_11_H_12_O_7_	Phenylpropanoic acids
Saquinavir	9.705	669.3774	669.3770	0.582	[M−H]−	C_38_H_50_N_6_O_5_	Quinoline carboxamides
7‐(2‐hydroxypropan‐2‐yl)‐1,4a‐dimethyl‐2,3,4,5,6,7,8,8a‐octahydronaphthalen‐1‐ol	10.645	239.1375	239.1378	−1.333	[M−H]−	C_15_H_28_O_2_	Terpenes
5‐[(Z)‐12‐(3,5‐dihydroxyphenyl)dodec‐8‐enyl]benzene‐1,3‐diol	11.386	383.2228	383.2228	0.052	[M−H]−	C_24_H_32_O_4_	Resorcinols
5‐(8,11,14‐pentadecatrienyl)resorcinol	13.151	313.2523	313.2520	0.924	[M−H]−	C_21_H_30_O_2_	Resorcinols

### 3.2 Body length and head swing

Statistical analysis revealed no significant differences in body length or head swing between the 0.5 mg/mL sweet pepper group and the control group. However, compared to the control group, body lengths in the 0.75 mg/mL and 1 mg/mL sweet pepper groups significantly increased from 942.57 ± 80.72 μm to 1049.2 ± 62.23 μm and 1046.13 ± 89.77 μm, respectively (*p* < .01) (Figure 1b). Additionally, head swing also significantly increased from 14.1 ± 3.1 times per minute to 22.3 ± 3.4 times and 26.6 ± 1.7 times per minute in these groups, respectively (*p* < .01) (Figure 1c).

During the larval stage, the nervous system of *C. elegans* is not fully developed, making them sensitive to environmental changes. Pepper extract was administered to *C. elegans* starting from the L1 stage. Upon reaching adulthood, the pepper extract‐treated *C. elegans* showed no significant differences in body length or locomotion compared to the untreated controls, indicating that the sweet pepper extract did not adversely affect their growth and development.

### 3.3 Sweet pepper reduced glucose and lipid levels in *C. elegans*

**FIGURE 2:**
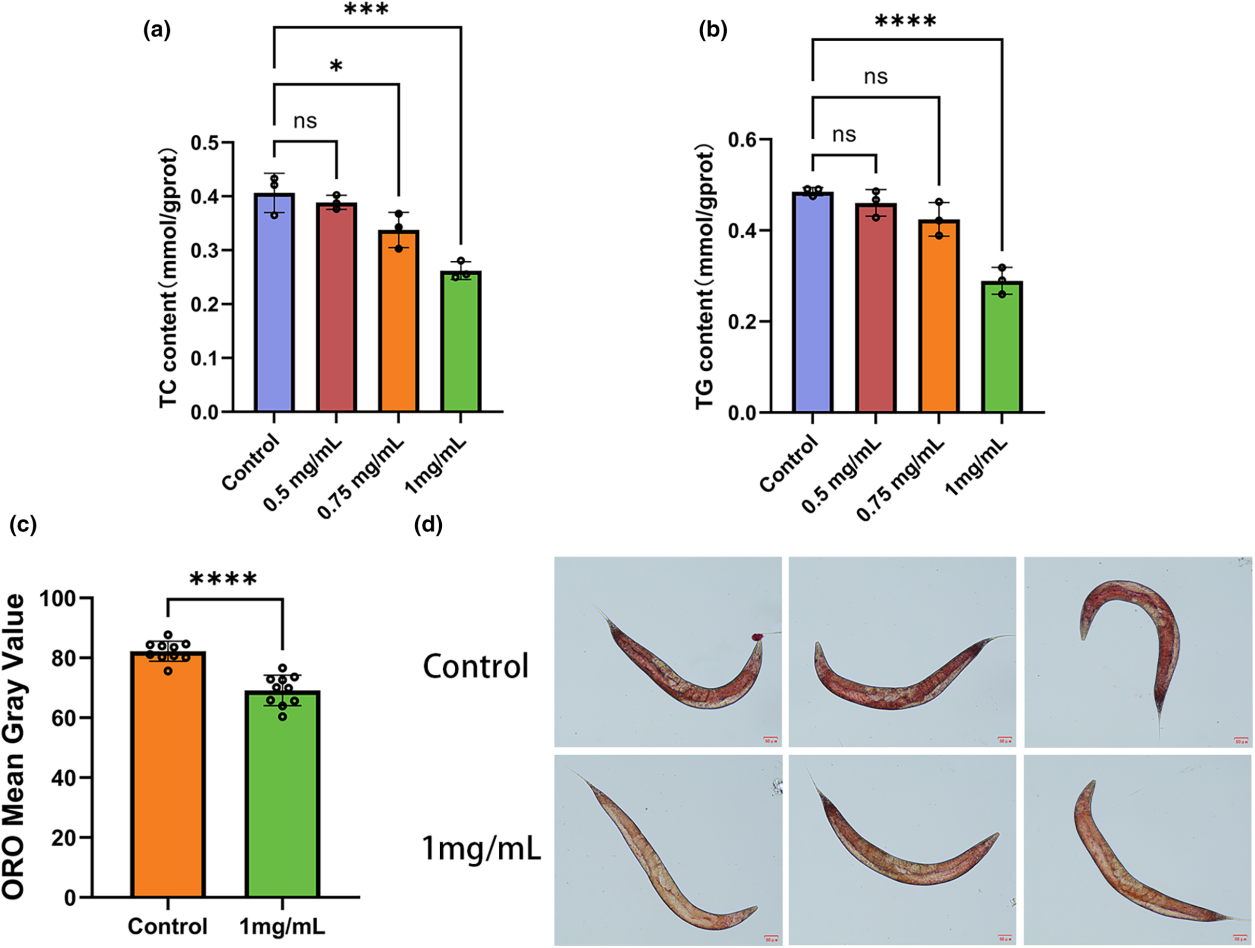
Sweet pepper reduced lipid levels in *C. elegans*. (a) The TC content of *C. elegans* (*n* = 3). (b) The TG content of *C. elegans* (*n* = 3). (c) ORO staining means gray value of *C. elegans* (*n* = 10). (d) Representative images of *C. elegans* stained with oil red O. Statistical analysis was conducted using Student's *t*‐test. Data are shown as mean ± SD. *p < .05, ***p < .001, ****p < .0001, ns, no significance.

Compared to the control group, the glucose levels in the 0.5 mg/mL, 0.75 mg/mL, and 1 mg/mL sweet pepper groups decreased from 5.49 ± 0.29 μmol/mgprot to 3.40 ± 0.43 μmol/mgprot (*p* < .05), 3.04 ± 0.29 μmol/mgprot (*p* < .01), and 1.11 ± 0.13 μmol/mgprot (*p* < .01), respectively (Figure 1d). The lipid analysis revealed that TC content decreased from 0.41 ± 0.04 mmol/gprot to 0.26 ± 0.02 mmol/gprot with 1 mg/mL sweet pepper treatment (*p* < .01). However, 0.5 mg/mL sweet pepper did not produce a significant decrease in TC levels in *C. elegans* (Figure 2a). After treatment with 1 mg/mL sweet pepper, the TG content decreased from 0.49 ± 0.01 mmol/gprot to 0.29 ± 0.03 mmol/gprot (*p* < .01). No significant differences were observed in the 0.5 mg/mL and 0.75 mg/mL groups compared with the control group (Figure 2b). Oil red O staining results showed that 1 mg/mL sweet pepper significantly reduced the number of lipid droplets and the relative area of staining (Figure 2c,d). These results suggest that sweet pepper extract significantly reduces the levels of TC, TG, and glucose in *C. elegans*, contributing to suppressed fat accumulation.

### 3.4 RNA‐seq and enrichment analysis

**FIGURE 3:**
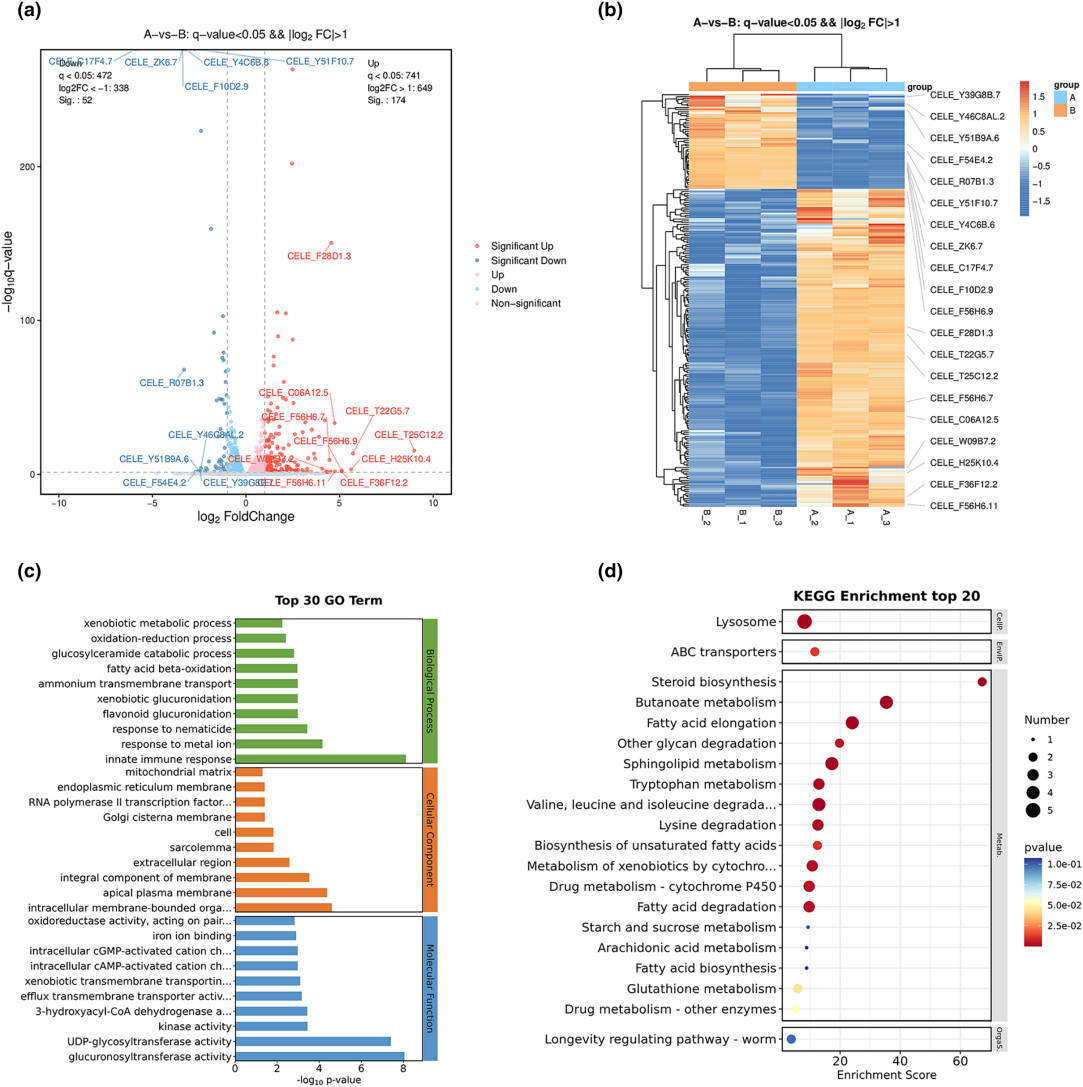
RNA‐seq and enrichment analysis. A: 1.0 mg/mL sweet pepper group. B: Control group. (a) Volcano plot of significant DEGs (FC > 1.5, *p* < .05). (b) DEGs orange represents upregulation, while blue represents downregulation (FC > 1.5, *p* < .05). (c) GO analysis of DEGs expression. (d) KEGG analysis of DEGs. The size of the circles corresponds to the number of DEGs and are color coded according to −log_10_ (*p* value). The *x*‐axis shows the enrichment factor value.

RNA‐seq analysis was further performed to explore the potential mechanism of sweet peppers in reducing fat accumulation and glucose levels in *C. elegans*. Compared to the control group, 226 differentially expressed genes (DEGs) were identified in *C. elegans* after treatment with sweet pepper, comprising 174 upregulated and 52 downregulated genes (Figure 3a,b). The Gene Ontology (GO) enrichment analysis revealed that 42 biological processes (BP) entries were predominantly linked to the innate immune response, fatty acid beta‐oxidation, and response to metal ion. Additionally, 43 molecular function (MF) entries were identified, primarily associated with activities such as glucuronosyltransferase, UDP‐glycosyltransferase activity, iron ion binding, structural constituent of cuticle, and heme binding. Additionally, 12 cellular components (CC) entries were mainly linked to collagen trimer, intracellular membrane‐bounded organelle, extracellular region, endoplasmic reticulum membrane, and collagen‐ and cuticulin‐based cuticle extracellular matrix (Figure 3c). Furthermore, KEGG pathway enrichment analysis further highlighted the alteration of 25 related signaling pathways, significantly enriching pathways involved in the biosynthesis of unsaturated fatty acids, fatty acid elongation, ABC transporters, lysosome, and sphingolipid metabolism (Figure 3d). These findings suggest that sweet pepper affects key metabolic and cellular pathways that are crucial for maintaining cellular structure and metabolism in *C. elegans*.

### 3.5 Untargeted metabolomics analysis

**FIGURE 4:**
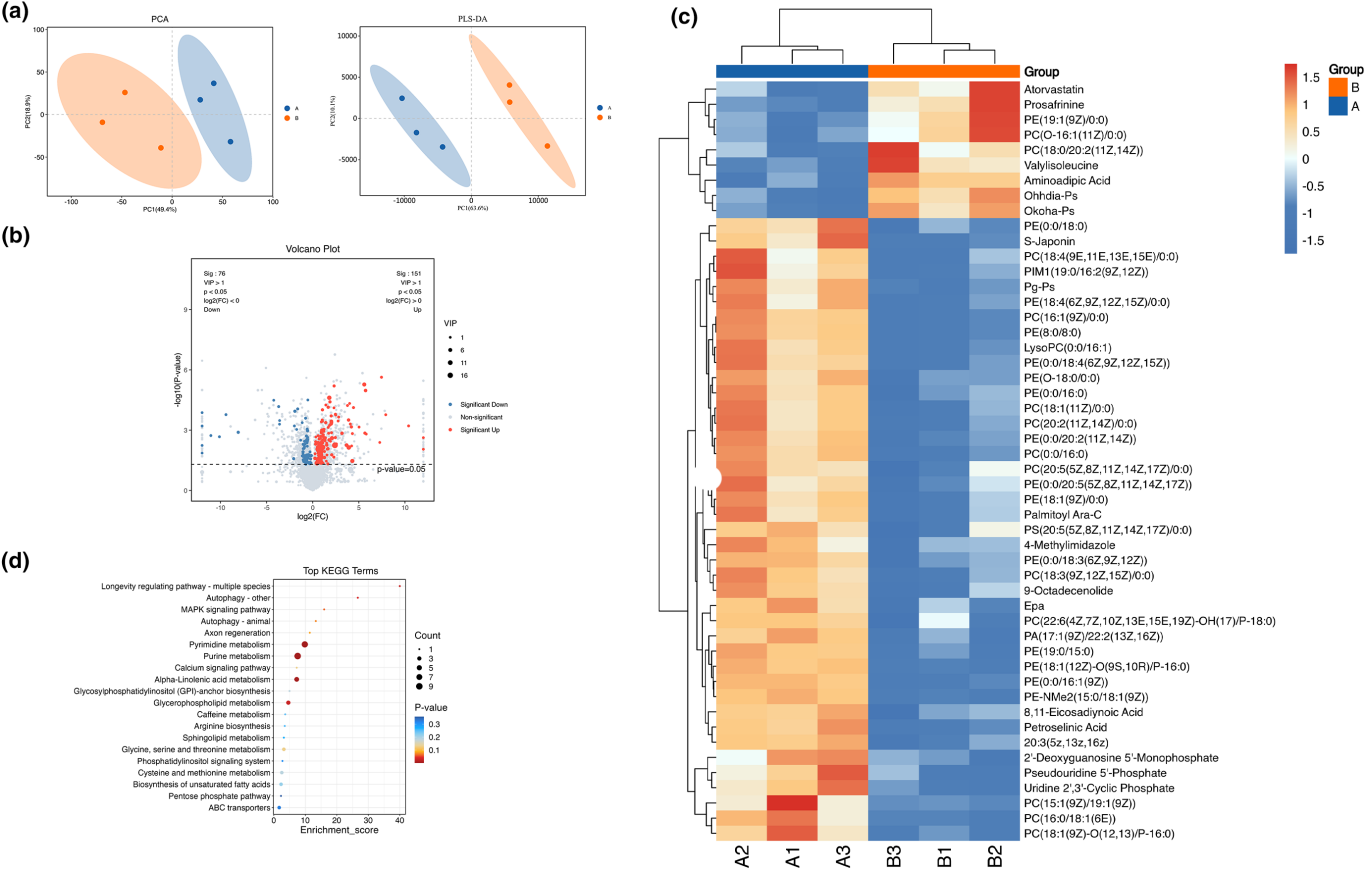
Identification of differential metabolites and enrichment analysis of metabolic pathways. A: 1 mg/mL sweet pepper. B: Control. (a) Principal component analysis (PCA) and partial least squares discriminant analysis (PLS‐DA). (b) Volcano plot of differential metabolites. (c) Heatmap of differential metabolites. (d) KEGG pathway analysis of differential metabolites. The size of the circles corresponds to the number of DEMs and is color coded according to *p* value.

The metabolic impact of 1 mg/mL sweet pepper on *C. elegans* was investigated through untargeted metabolomics. The analysis revealed distinct clustering between the 1 mg/mL sweet pepper group and the control group, indicating significant metabolic changes (Figure 4a). A total of 227 differential metabolites were identified based on the criteria of VIP >1 and *p* < .05, consisting of 151 upregulated and 76 downregulated metabolites (Figure 4b). Heatmap analysis highlighted a marked increase in the levels of fatty acids and phospholipids following treatment with sweet pepper (Figure 4c). Further metabolic pathway enrichment analysis of these differential metabolites revealed the modulation of multiple metabolic pathways, including ABC transporters, pentose phosphate pathway, biosynthesis of unsaturated fatty acids, cysteine and methionine metabolism, phosphatidylinositol signaling system, metabolism of glycine, serine, and threonine, sphingolipid metabolism, arginine biosynthesis, caffeine metabolism, glycerophospholipid metabolism, glycosylphosphatidylinositol (GPI)‐anchor biosynthesis, alpha‐linolenic acid metabolism, calcium signaling pathway, purine metabolism, pyrimidine metabolism, axon regeneration, autophagy in animals, MAPK signaling pathway, other forms of autophagy, and longevity‐regulating pathways across multiple species (Figure 4d). These pathways suggest that sweet pepper may influence essential metabolic and signaling pathways that contribute to its effects on fat accumulation and longevity in *C. elegans*.

### 3.6 Integration of gene and metabolite networks in *C. elegans*

**FIGURE 5:**
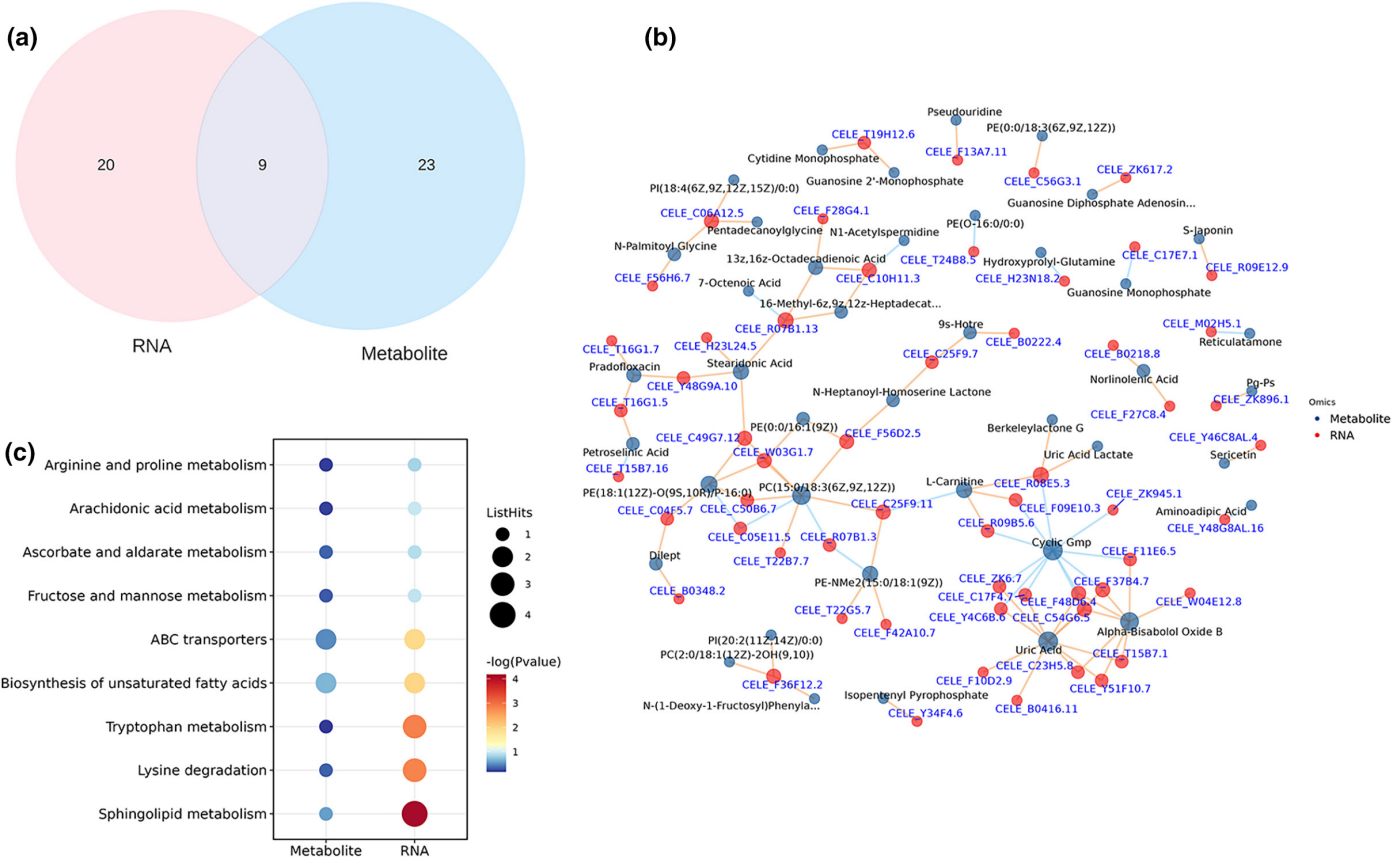
Metabonomic and transcriptome integration analysis. (a) Venn diagram of differential genes pathway and metabolites pathway. (b) Network plot of differential genes and metabolites. (c) Common differential pathways.

As depicted in Figure 5a,b, we further constructed a differential network integrating significantly altered genes and metabolites, subsequently mapping these entities to their corresponding pathways. As a result, nine commonly affected pathways were identified (Figure 5c): arginine and proline metabolism, arachidonic acid metabolism, ascorbate and aldarate metabolism, fructose and mannose metabolism, ABC transporters, biosynthesis of unsaturated fatty acids, tryptophan metabolism, lysine degradation, and sphingolipid metabolism. The analysis revealed significant modifications in multiple amino acid metabolic pathways in *C. elegans* following sweet pepper treatment. Amino acids are crucial for the function of numerous enzymes, and alterations in their metabolism can significantly affect various biological pathways. Therefore, changes in amino acid metabolism are likely contributory factors to the observed reduction in fat content in *C. elegans* after sweet pepper treatment, as indicated by recent studies (Liu et al., 2023; Nanda et al., 2023). Additionally, we noted a pronounced enrichment in the biosynthesis pathway of unsaturated fatty acids. Previous research has indicated a significant correlation between the biosynthesis pathway of unsaturated fatty acids and the accumulation of fat in *C. elegans* (Ding et al., 2015).

### 3.7 Sweet pepper inhibited the expression levels of the SBP‐1 and SCD1 genes

**FIGURE 6:**
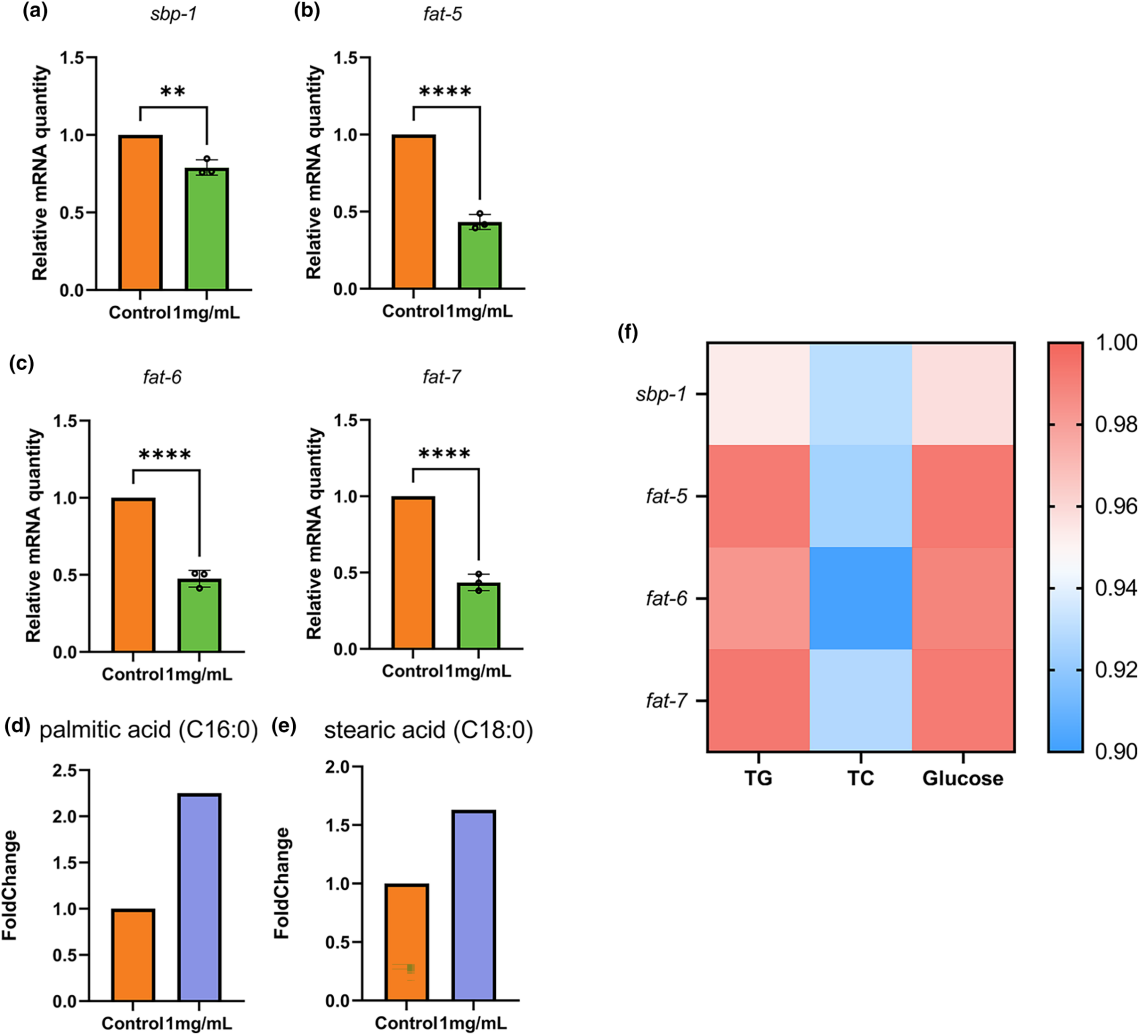
Effect of sweet pepper on SBP‐1 and SCD1 gene expression. (a) The sbp‐1 mRNA levels in *C. elegans*. (b) The fat‐5 mRNA levels in *C. elegans*. (c) The fat‐6 and fat‐7 mRNA levels in *C. elegans*. (d) Log (FC) of palmitic acid. (e) Log (FC) of stearic acid. (f) The Pearson correlation coefficient between gene expression and physiological indicators. Statistical analysis was conducted by Student's *t*‐test. Data are shown as mean ± SD. **p < .01, ****p < .0001, ns, no significance.

The expression levels of unsaturated fat biosynthesis rate‐limiting enzymes including *fat‐5* (delta(9)‐fatty‐acid desaturase *fat‐5*); *fat‐6* (delta(9)‐fatty‐acid desaturase *fat‐6*); and *fat‐7* (delta(9)‐fatty‐acid desaturase *fat‐7*) were detected. The results demonstrated that 1 mg/mL sweet pepper significantly reduced the gene expression levels of *fat‐5*, *fat‐6*, and *fat‐7* in *C. elegans* (Figure 6b,c). Additionally, there was a significant decrease in the mRNA expression level of the regulatory component *sbp‐1* for these genes (Figure 6a). Furthermore, treatment with 1 mg/mL sweet pepper resulted in increased levels of unsaturated fatty acids, such as palmitic acid and stearic acid (Figure 6d,e).

To further substantiate the reduction in fat accumulation caused by sweet pepper extract, we conducted a correlation analysis between target genes (*sbp‐1*, *fat‐5*, *fat‐6*, and *fat‐7*) and biochemical markers (glucose, TG, and TC). The outcome showed that after receiving 1 mg/mL of sweet pepper extract, target genes substantially associated with biochemical indicators. Specifically, we observed a correlation coefficient of 0.95 between the target gene sbp‐1 and triglycerides (TG), and a correlation coefficient of 0.93 with TC. Additionally, genes related to fat metabolism (*fat‐5*, *fat‐6*, and *fat‐7*) exhibited notable positive correlations with the *sbp‐1* (Figure 6f).

### 3.8 Sweet pepper decreases fat accumulation through the SREBP‐SCD axis

**FIGURE 7:**
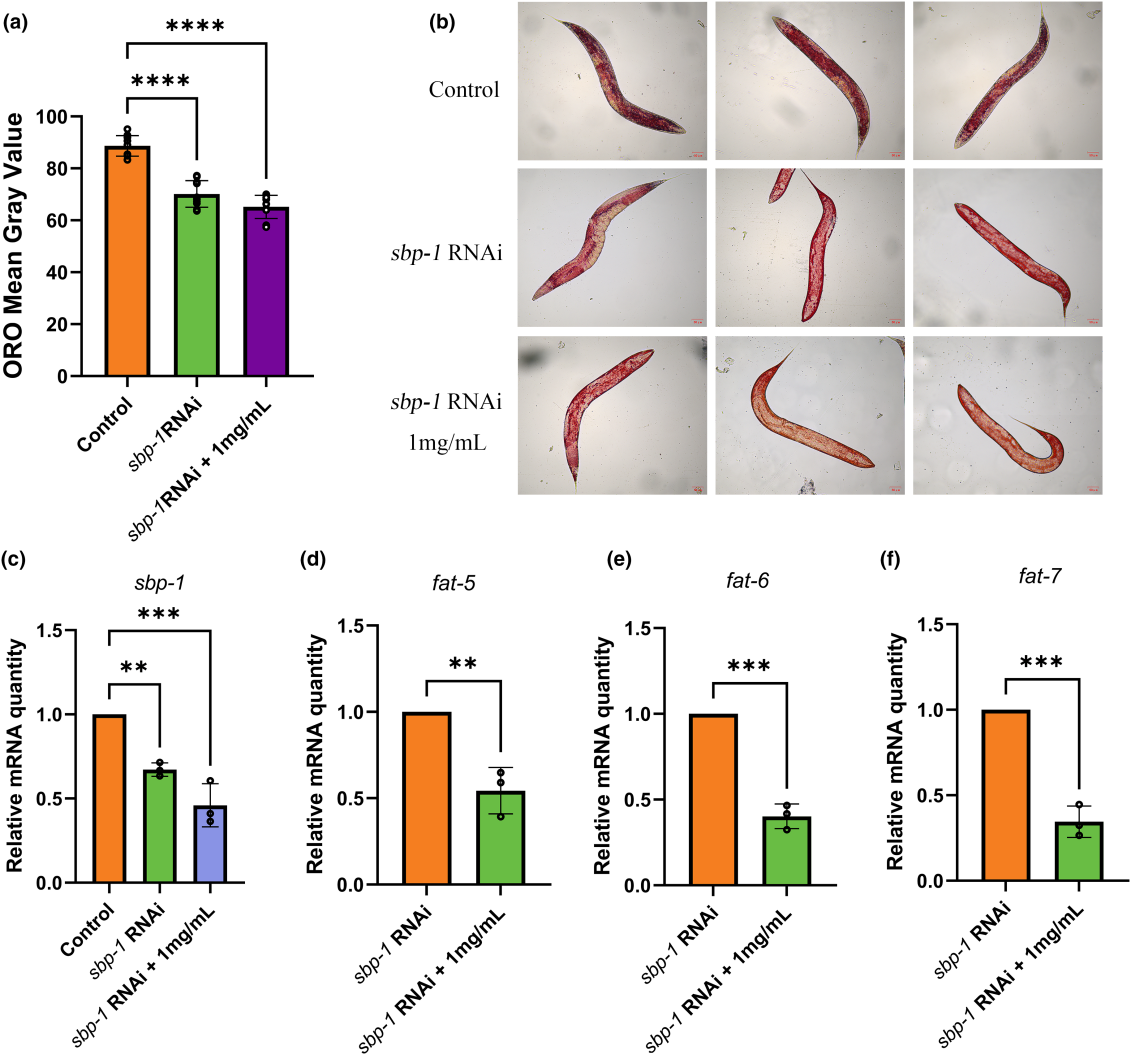
Effect of sweet pepper on *C. elegans* after knocking down sbp‐1. (a) ORO staining means gray value of *C. elegans*. (b) Representative images of *C. elegans* stained with oil red O (*n* = 10). (c) The sbp‐1 mRNA levels in *C. elegans*. (d) The fat‐5 mRNA levels in *C. elegans* (*n* = 3). (e) The fat‐6 mRNA levels in *C. elegans* (*n* = 3). (f) The fat‐7 mRNA levels in *C. elegans* (*n* = 3). Statistical analysis was conducted by Student's *t*‐test. Data are shown as mean ± SD. **p < .01, ***p < .001, ****p < .0001, ns, no significance.

The oil red O positive staining area of *C. elegans* in the *sbp‐1* RNAi group and *sbp‐1* RNAi +1 mg/mL sweet pepper group was significantly lower than that in control group (Figure 7a,b). The result of epistasis experiment showed that 1 mg/mL sweet pepper further significantly downregulated the mRNA expression of *sbp‐1* in *C. elegans* with *sbp‐1* gene knockdown (Figure 7c). Furthermore, compared with the *sbp‐1* RNAi group, 1 mg/mL sweet pepper significantly decreased the mRNA levels of *fat‐5*, *fat‐6*, and *fat‐7* in *C. elegans* (Figure 7d–f).

## 4 DISCUSSION

With the shift in lifestyle and dietary habits, obesity has become a significant concern. Prolonged lipid accumulation serves as a precursor to various ailments, including metabolic disorders and a spectrum of CVDs. In severe cases, these conditions may even lead to fatalities (Natesan & Kim, 2021). Long‐term nutritional imbalance and fat accumulation serve as a primary contributor to obesity. Acting as the hub of nutrient sensing and autophagy, lysosomes are instrumental in the breakdown of cellular lipids. Notably, when lysosomal function is compromised, there is a significant reduction in both basal and nutrient‐induced fat accumulation in *C. elegans*. This underscores the pivotal role of lysosomes in lipid metabolism and obesity (Lu et al., 2022). Researchers typically employ Nile red or BODIPY‐labeled fatty acids to study fat accumulation. However, certain studies have indicated that lysosome‐related organelles, marked with Nile red and BODIPY, do not represent the primary fat accumulation sites in *C. elegans*. Through the verification of biochemical indicators, researchers have found that oil red O staining is an effective method for assessing the main fat accumulation in nematodes (O'Rourke et al., 2009). Sweet pepper, as a natural plant source, has consistently been proven across various studies to exhibit certain beneficial effects in the context of obesity and T2DM (Liang et al., 2023; Park et al., 2014). In this study, metabolomics and RNA‐seq analysis were employed to explore the relationship between the reduction of fat accumulation, glucose content, and SREBP‐SCD axis in sweet pepper in *C. elegans*. The results revealed that a newly discovered sweet pepper, initially named Jinjiao 23–6, effectively inhibits α‐glycosidase activity, reduces glucose levels, and significantly lowers lipid content, including TG and TC, without adversely affecting its growth and development. Additionally, we found that the biosynthesis of unsaturated fatty acids and ABC transporters signaling pathways were well enriched in both metabolomics and transcriptomics. Moreover, the biosynthesis rate‐limiting enzymes for unsaturated fatty acids, SBP‐1 and SCD1 (*fat‐5*, *fat‐6*, and *fat‐7*), were significantly downregulated after treatment with sweet pepper.

In our study, we initially characterized the primary and secondary metabolites present in the newly discovered sweet pepper (Jinjiao 23‐6). Subsequently, we observed a significant reduction in fat content within the nematodes treated with 1 mg/mL sweet pepper extract, leading to decreased levels of TG, TC, and glucose, without substantial effects on their growth and development. These results are consistent with the findings of Alexander V Sirotkin et al, who reported similar effects of pepper on fat accumulation reduction and its potential health benefits (Sirotkin, 2023). And they also demonstrated that sweet pepper has a notable impact on reducing blood sugar and inhibiting fat accumulation.

Furthermore, to uncover the potential mechanism by which sweet pepper reduces intestinal fat accumulation in *C. elegans*, metabolomics and transcriptomics were employed to analyze potential differentially expressed genes and metabolites, and to reveal their mechanisms of action. Through a multiomics approach, the notably downregulated genes were enriched in GO analysis, specifically in stearoyl‐CoA 9‐desaturase activity (SCD1). Stearoyl‐CoA desaturase (SCD1) is a rate‐limiting enzyme involved in catalyzing the production of unsaturated fatty acids (Dziewulska et al., 2020). *C. elegans*, unlike mammals, possesses the ability to synthesize unsaturated fatty acids de novo and has seven fatty acid desaturases (Watts, 2009). In its system, there exist three Δ9 desaturases: *fat‐5*, *fat‐6*, and *fat‐7*. Among them, *fat‐5* desaturase exhibits specificity toward palmitic acid (C16:0), whereas *fat‐6* and *fat‐7* primarily act on stearic acid (C18:0) (Shen et al., 2018).

In *C. elegans*, our focus shifted toward the genes that encode the SCD enzymes, namely *fat‐5*, *fat‐6*, and *fat‐7*. We observed a significant decrease in the expression levels of these genes. This correlated with the noticeable increase in stearic acid (C18:0) and palmitic acid (C16:0) levels identified via metabolomics analysis (Figure 6d,e). Similarly, Jibin Kim and colleagues showed that a mixture of green tea and java pepper had an antiobesity effect by reducing the expression levels of SCD1 (Kim et al., 2023). All these findings suggest that sweet pepper inhibits SCD1, including *fat‐5*, *fat‐6*, and *fat‐7*, which increase the levels of stearic acid (C18:0) and palmitic acid (C16:0) to reduce fat accumulation.

Additionally, it is worth mentioning that mRNA expression of SREBP homolog SBP‐1 was also significantly downregulated in *C. elegans* after treatment with sweet pepper. Activated SBP‐1 transmits from the cytoplasm to the nucleus, where it transcribes and activates SCD1, which mediates biosynthesis of unsaturated fatty acids. Inhibition of the SREBP‐SCD axis has been shown to reduce lipid accumulation in *C. elegans* (Zhang et al., 2017). When *sbp‐1* was knocked down, the effect of sweet pepper extract on reducing fat content of *C. elegans* was significantly weakened. Hence, we propose that sweet pepper alleviates lipid accumulation by modulating the synthesis of unsaturated fatty acids through SREBP‐SCD axis.

Several shortcomings still exist in this study. Firstly, it only demonstrates the downregulating effect of sweet pepper on the SREBP‐SCD axis, modifying unsaturated fatty acid biosynthesis and reducing fat accumulation, without delving into the mechanism through which sweet pepper regulates this axis. Secondly, while we have identified the primary and secondary metabolites of sweet pepper (Jinjiao 23–6) for the first time, we have not established any potential relationship between these metabolites and *sbp‐1* and SCD1. Last but not least, further investigation is required to thoroughly explore the impact of sweet pepper on T2DM.

Overall, the new germplasm pepper can exert the effect of fat lowering in *C. elegans*. It has the potential for the development of weight loss functional foods. Considering its advantages such as easy planting, high yield, and low cost, it may offer significant benefits to overweight population.

## AUTHOR CONTRIBUTIONS

**Junyi Wang:** Data curation (equal); investigation (equal); methodology (equal); resources (equal); validation (equal); visualization (equal); writing – original draft (equal). **Peng Xu:** Data curation (equal); methodology (equal); supervision (equal); validation (equal); visualization (equal); writing – original draft (equal). **Xinhua Liu:** Conceptualization (equal); formal analysis (equal); funding acquisition (equal); project administration (equal). **Chunxin Cao:** Conceptualization (equal); resources (equal). **Yingkun Sheng:** Supervision (equal); writing – review and editing (equal). **Jianfeng Wang:** Data curation (equal); funding acquisition (equal); methodology (equal); project administration (equal); writing – review and editing (equal).

## FUNDING INFORMATION

This work was supported by A Project Supported by the Scientific Research Fund of Zhejiang Provincial Education Department Y202147287 and Jinhua City Science and Technology Plan Key Project under Grant 2021‐2‐034.

## CONFLICT OF INTEREST STATEMENT

The authors declare no conflicts of interest.

## Supporting information


Appendix S1

## Data Availability

The raw sequence data have been submitted to the NCBI Short Read Archive (SRA) with accession number <PRJNA1067303>. The metabolomics raw data have been uploaded to the MetaboLights project number <MTBLS9326> URL www.ebi.ac.uk/metabolights/MTBLS9326 (Yurekten et al., 2024). The original contributions presented in the study are included in the article/Supplementary Material; further inquiries can be directed to the corresponding authors.
