# Supplementary material for: Sweet pepper extract reduces fat storage in Caenorhabditis elegans by SREBP‐SCD axis based on multiomics analysis
Source: Food Sci Nutr. 2024 Jun 14;12(9):6284–97. doi: 10.1002/fsn3.4266 (PMC11561784; doi:10.1002/fsn3.4266)
Supplement: Supplementary file 1 — Appendix S1 [file FSN3-12-6284-s001.docx]

**Figure S1: We measured the α-glucosidase inhibition rates of 27 mutagenized sweet pepper plants and selected the one with the highest inhibition rate as the parent for the breeding experiment.** **P1 represents pepper 1, and so on up to pepper 27.**

**
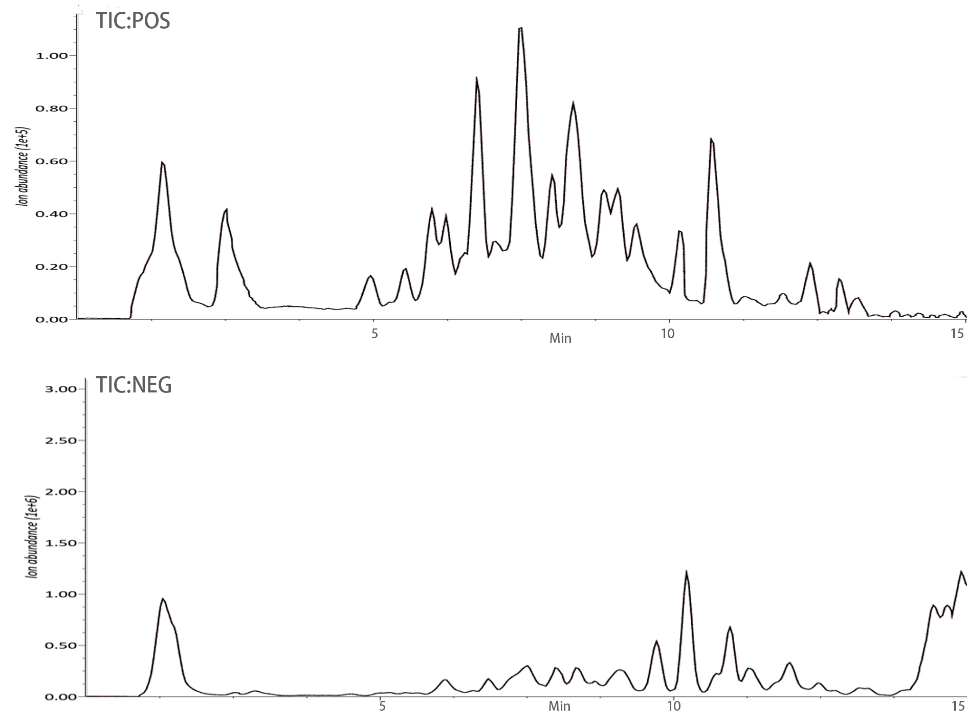
**

**Figure S2: Due to issues with column residue, the identification of substances became unfeasible after fifteen minutes. Therefore, chromatograms for the first 15 minutes are presented.**

**Table S1** The primers used for real-time PCR

| Gene name | Primer |
| --- | --- |
| *sbp-1* F | GGCGGCGAAGATTGTGATTC |
| *sbp-1* R | CGCTCGGTTTTTGGTCTTCG |
| *fat-5* F | GGGCTACAGTTGGATGGGTATT |
| *fat-5* R | GAGGGTGGCTTTGTAGGCTC |
| *fat-6* F | AGCGCTGCTCACTATTTCGG |
| *fat-6* R | AGTTGTGACCTCCCTCTCCG |
| *act-1* F | CTTGCCCCATCAACCATGA |
| *act-1* R | CAAGTTGACGAAGTTGTGCAT |
| *fat-7* F | CAAACGGCCGTCTTCTCATT |
| *fat-7* R | TCTCATTGGTGTGGTTGCCT |
